# Supplementary material for: MetaRibo-Seq measures translation in microbiomes
Source: Nat Commun. 2020 Jun 29;11:3268. doi: 10.1038/s41467-020-17081-z (PMC7324362; doi:10.1038/s41467-020-17081-z)
Supplement: Supplementary file 10 — Supplementary Data 7 [file 41467_2020_17081_MOESM10_ESM.zip › File2/Confidence_High_Taxonomy/367360_out.krona.html]

Javascript must be enabled to view this page.

members
magnitude
magnitudeUnassigned
count
unassigned
taxon
rank

367360\_out

20

2
20
superkingdom

201174
phylum
1

1
class
84998

1
order
1643822

1
species

SRS014923\_contig\_number\_35310
2137879

1239
19
phylum

186801
19
class

186802
order
19

family
19
186803

genus
19
841

166486
19
species

SRS011084\_contig\_number\_25967SRS014948\_contig\_number\_21511SRS015890\_contig\_number\_25040SRS017821\_contig\_number\_20860SRS018623\_contig\_number\_15092SRS019267\_contig\_number\_contig-100\_1502.46786SRS021484\_contig\_number\_28890SRS024549\_contig\_number\_910SRS049900\_contig\_number\_19813SRS050422\_contig\_number\_36592SRS050941\_contig\_number\_17722SRS1055022\_contig\_number\_23352SRS144183\_contig\_number\_20204SRS147271\_contig\_number\_31718SRS148091\_contig\_number\_12295SRS148721\_contig\_number\_42491SRS148784\_contig\_number\_13142SRS149879\_contig\_number\_27066SRS893252\_contig\_number\_contig-100\_1688.94495
